# A Digital Behavioral Weight Gain Prevention Intervention in Primary Care Practice: Cost and Cost-Effectiveness Analysis

**DOI:** 10.2196/12201

**Published:** 2019-05-17

**Authors:** Anirudh Krishnan, Eric Andrew Finkelstein, Erica Levine, Perry Foley, Sandy Askew, Dori Steinberg, Gary G Bennett

**Affiliations:** 1 Program in Health Services and Systems Research Duke-NUS Medical School Singapore Singapore; 2 Duke Global Digital Health Science Center Duke University Durham, NC United States

**Keywords:** cost-effectiveness, cost-benefit analysis, obesity, telemedicine, women’s health, minority health, weight gain prevention, weight gain

## Abstract

**Background:**

Obesity is one of the largest drivers of health care spending but nearly half of the population with obesity demonstrate suboptimal readiness for weight loss treatment. Black women are disproportionately likely to have both obesity and limited weight loss readiness. However, they have been shown to be receptive to strategies that prevent weight gain.

**Objective:**

The aim of this study was to evaluate the costs and cost-effectiveness of a digital weight gain prevention intervention (Shape) for black women. Shape consisted of adaptive telephone-based coaching by health system personnel, a tailored skills training curriculum, and patient self-monitoring delivered via a fully automated interactive voice response system.

**Methods:**

A cost and cost-effectiveness analysis based on a randomized clinical trial of the Shape intervention was conducted from the payer perspective. Costs included those of delivering the program to 91 intervention participants in the trial and were summarized by program elements: self-monitoring, skills training, coaching, and administration. Effectiveness was measured in quality-adjusted life years (QALYs). The primary outcome was the incremental cost per QALY of Shape relative to usual care.

**Results:**

Shape cost an average of US $758 per participant. The base-case model in which quality of life benefits decay linearly to zero 5 years post intervention cessation, generated an incremental cost-effectiveness ratio (ICER) of US $55,264 per QALY. Probabilistic sensitivity analyses suggest an ICER below US $50,000 per QALY and US $100,000 per QALY in 39% and 98% of simulations, respectively. Results are highly sensitive to durability of benefits, rising to US $165,730 if benefits end 6 months post intervention.

**Conclusions:**

Results suggest that the Shape intervention is cost-effective based on established benchmarks, indicating that it can be a part of a successful strategy to address the nation’s growing obesity epidemic in low-income at-risk communities.

## Introduction

Excess weight is estimated to account for 9% of total annual health care costs, with roughly half paid by public sector health programs [1]. Nationally, almost 55% of black women have obesity compared with 38% of white women [2]. As a result, black women are at greater risk for obesity-related chronic diseases, including stroke, coronary heart disease, and depression, and attributable costs [3,4]. As a result, interventions that successfully address excess weight in this at-risk group may confer significant health and economic benefits to individuals and society. However, reducing risk factors in this group is a challenge because, relative to other populations, black women express less interest in or readiness for weight loss treatment [5,6]. Moreover, weight loss interventions have shown consistently smaller weight loss among black women relative to their white counterparts [7-10]. Therefore, delivering interventions that seek to prevent weight gain, as opposed to promoting weight loss, might be a more successful treatment strategy [11].

Weight gain prevention strategies align with sociocultural norms among black communities that are tolerant of higher body weights [12-14]. Previous digital weight gain prevention interventions have shown moderate success in reducing weight gain among children and young adults with overweight, but no studies had studied their effectiveness among black women [15-17]. Bennett et al developed *The Shape Program* to test a tailored digital health solution aimed at helping black women prevent weight gain [18,19]. Results reveal Shape’s effectiveness in preventing weight gain among black women. However, whether Shape is cost-effective remains unknown; that is the focus of this analysis.

A cost-effectiveness analysis is one strategy for understanding whether the benefits of an intervention are worth the costs. Many public sector agencies, such as the National Institute for Health and Care Excellence in the United Kingdom and the Health Intervention and Technology Assessment Program in Thailand, require cost-effectiveness analyses before considering a subsidy decision for a health intervention [20]. Although the United States does not systematically require cost-effectiveness analyses, they have gained popularity as a tool to compare the value of diverse interventions. Guidelines recommend that cost-effectiveness analyses report benefits in terms of a common metric such as the quality-adjusted life year (QALY), which consolidates diverse health benefits to facilitate comparisons of value among interventions targeting diverse population health gaps [21,22]. In this study, we present the costs and cost-effectiveness of Shape relative to usual care (UC) in terms of cost per QALY gained and compare this value to established benchmarks for cost-effectiveness. Given the risk of steady weight gain in the target population, third-party payers may be interested in knowing whether a successful weight gain prevention program, such as Shape, represents good use of scarce health care resources.

## Methods

### The Shape Program

The Shape Program (Shape) was designed to prevent weight gain in black female primary care patients whose body mass index (BMI) placed them in either the overweight (25 to 29.9 kg/m^2^) or class 1 obese (30 to 34.9 kg/m^2^) categories. Shape sought to promote the modification of obesogenic lifestyle behaviors (diet, physical activity, and leisure time activities). It leveraged key technological innovations to support personnel within a private, nonprofit community health center network [19]. In doing so, the program was able to augment the capacity of existing health systems to reach patients who otherwise would receive little or no weight management counseling. The Shape program included adaptive telephone-based coaching by health system personnel, personalized obesogenic behavior change goals assigned every 2 months, a tailored skills training curriculum, patient self-monitoring delivered via a fully automated interactive voice response system, 12 counseling calls with a registered dietitian, and a 12-month gym membership [19].

Shape’s effectiveness relative to a light touch UC intervention was tested among 194 overweight and class 1 obese black women aged 25 to 44 years in a 2-arm parallel-group randomized controlled trial over 12 months followed by a 6-month follow-up period (ClinicalTrials.gov reference: NCT00938535) [18]. Additional inclusion criteria were having visited a member in the health center in the past 24 months, being a state resident, and being fluent in English. Participants were excluded if they were pregnant, up to 12 months postpartum, had a myocardial infarction or stroke in the past 2 years, or had any history of cognitive, developmental, or psychiatric disorders. In the trial, UC consisted of the *Aim for a Healthy Weight* brochure and semiannual newsletters on health topics not related to weight. Intent-to-treat analyses included outcome measurements for 91 participants randomized to receive the intervention and 94 UC participants [18]. At 12 and 18 months, Shape participants had lower weight gain than UC participants (mean difference of −1.4 kg and −1.7 kg at 12 and 18 months, respectively).

### Cost Analysis

To estimate the incremental costs of Shape, we employed an activity-based costing method that links program resource consumption to specific program components [23,24]. This approach allows evaluators to map the resource flow of the program. Electronic budgetary records, staff interviews, and engagement data were utilized to estimate program costs. All costs were inflated to 2018 US dollars using the medical portion of the seasonally adjusted US consumer price index [25].

### Cost-Effectiveness Analysis

The cost-effectiveness analysis, which consisted of quantifying the incremental costs and QALYs of Shape relative to UC, was conducted from the third-party payer perspective.

#### Incremental Cost

As virtually no costs were incurred in the usual-care arm, the incremental cost is set equal to the cost of program delivery of the Shape intervention (including self-monitoring, skills training, coaching, and administration costs). This excluded program *development* costs, as these represent sunk costs that would not need to be repeated if the program were more broadly adopted. The average per capita cost of program delivery was assigned to the 91 participants who received the intervention and were included in the intent-to-treat analysis.

#### Incremental Effectiveness

The primary measure of effectiveness in the trial was weight change from baseline to 12 months. We converted the weight change into a health-related quality of life (QoL) change score over this time period. This imputation followed the regression approach described in Finkelstein and Kruger [26] using data from Finkelstein et al [27] and restricting it to a sample of women with a BMI between 25 and 35. This age and gender restriction allowed for obtaining estimates in a subsample that best approximates the characteristics of the Shape study population. Using this restricted sample, we estimated the association between QoL change and weight change (in kilograms) while controlling for baseline BMI and age via the following equation:

Δ QoL
_i_=β
_1_ × Δ weight
_i_ + β
_2_ × (Δ weight
_i_)
^2^ + β
_3_ × baseline BMI
_i_ + β
_4_ × baseline age
_i_ + ε
_i_

Using a process of step-wise regression, iteratively dropping variables found not to be statistically significant at the 5% significance level, we identified the following relationship:

Δ QoL=– 0.0029 × Δ weight + 0.0002 × baseline age

We used this equation to impute a QoL change for each individual in the Shape trial.

#### Cost-Effectiveness Analysis

As with the primary analysis, the cost-effectiveness analysis was based on the intention-to-treat sample, with missing observations in both trial arms treated as missing at random [18]. The numerator of the incremental cost-effectiveness ratio (ICER) is the incremental cost to deliver Shape. The denominator is the mean discounted QALYs gained by intervention participants minus mean discounted QALYs gained by the UC group. QALY estimates for each arm were generated by plotting a curve of ΔQoL against time from baseline and taking the area under this curve. All post-trial QALY estimates were discounted at 3.5% per annum.

In the base case, we used estimates of QoL change from baseline to each of 6 months, 12 months, and 18 months, and then assumed QoL benefits decay linearly until the end of the fifth year postcessation of the intervention, at which time we assume no further benefits.

#### Sensitivity Analyses

We assessed the sensitivity of our ICER to changes in key inputs using 1-way sensitivity analyses. We estimated the effect of the following changes on the ICER: (1) halving the cost of the intervention; (2) doubling and halving the costs incurred in each cost category; (3) doubling or halving the incremental effectiveness of the intervention with regard to UC; and (4) varying the duration of residual benefits post cessation from 5 years in the base case to 0.5 and 3 years.

#### Probabilistic Sensitivity Analyses

In addition, we conducted 10,000 simulations of the model to quantify the probability that the intervention is cost-effective for a range of willingness-to-pay thresholds that decision makers might consider. Cost was assumed to follow a gamma distribution, with an SD of 25% of mean costs; effectiveness was assumed to follow a normal distribution, with SDs equal to the SEs of effectiveness estimates.

The methods described in this section and reporting of results throughout the paper are consistent with the Consolidated Health Economic Evaluation Reporting Standards [21].

## Results

### Program Costs

The total cost of Shape was US $758 per participant in 2018 US dollars (Table 1) for the 1-year intervention. Program costs were allocated to 4 areas, including administration, self-monitoring, skills training, and counseling (Table 1). Administration costs, including personnel, costs of support staff training, and space and other overheads were the greatest consumer of program resources at an average cost of US $387 per participant. Telephone counseling costs were the second highest cost driver, driven largely by registered dietitians’ personnel costs and cell phone plan subscriptions, at an average of US $149 per participant. Interactive self-monitoring included server and interactive voice response system maintenance costs and purchasing of pedometers and scales and cost an average of US $126 per participant. Tailored skills training costs US $95 per participant, primarily driven by the cost of printing training materials and providing kit bags to participants. Training the coaches front-loaded many of the costs in the first 2 years of Shape. Specifically, the average program costs in years 1 and 2 (US $17,401) were 53% higher than the average costs in years 3 to 5 (US $11,380).

The variability in Shape coaching costs in years 1 and 2 was further explored. Shape coaches placed 3968 calls to participants (an average of 44 calls per participant during the yearlong program). The majority of these calls (3316/3968, 83.6%) were attempts to reach participants to deliver coaching content, while 16.4% (652/3968) were considered successful coaching calls in which the curriculum was delivered in full. These successful calls were on average 21.2 min long (SD 10.1 min). The average amount of time that coaches spent on unsuccessful calls per participant was 27.6 min (SD 19.2 min) for the whole program period.

**Table 1:** Program delivery costs for 91 participants by program area and year (all figures in 2018 US $).

Program area	Year 1 (US $)	Year 2 (US $)	Year 3 (US $)	Year 4 (US $)	Year 5 (US $)	Total (US $)	Cost per participant^a^ (US $)	Cost as percentage of total^b^
Interactive self-monitoring	7123	1140	1102	1068	1047	11,481	126	17%
Tailored skills training	1785	4585	2161	132	0	8662	95	13%
Telephone counseling	1877	3417	3301	3199	1794	13,588	149	20%
Administration	7665	7210	6967	6750	6619	35,212	387	51%
Total	18,450	16,352	13,531	11,148	9461	68,942	758	100%

^a^Calculated for a total of 91 intervention participants.

^b^Total does not sum to 100% due to rounding.

### Cost-Effectiveness Analysis

#### Incremental Cost

As virtually no cost was incurred in the UC arm, the incremental cost of Shape relative to UC was US $758 in the base case.

#### Effectiveness

**Figure 1:**
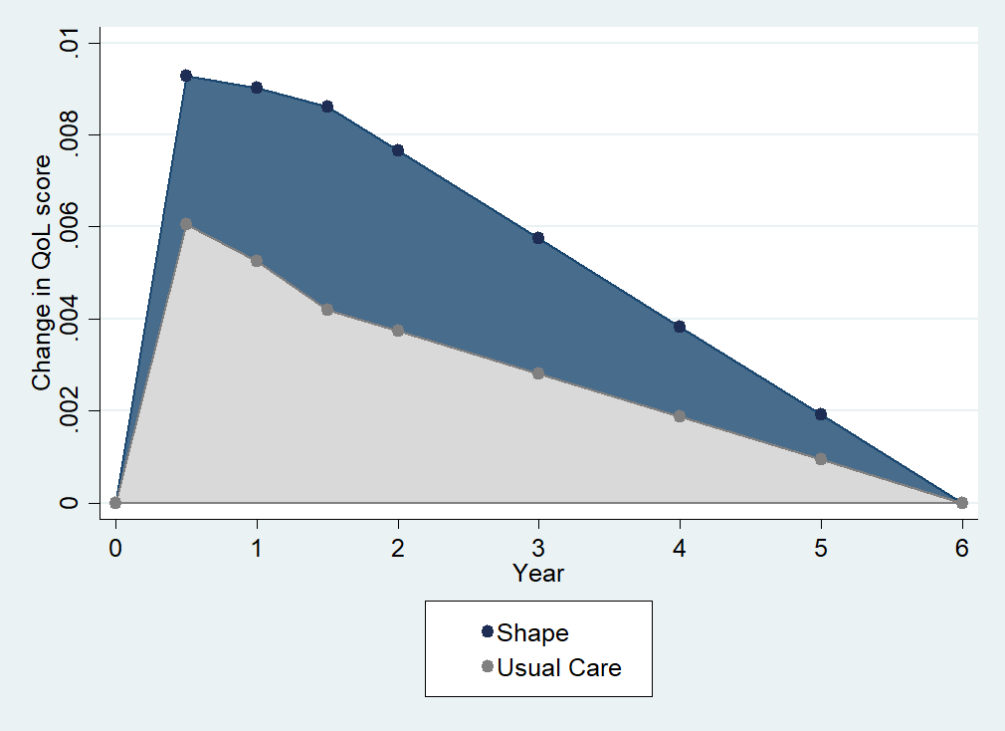
Estimated quality of life (QoL) change score plotted against time from baseline. The difference in area under the Shape curve and the Usual Care curve (ie, dark shaded region) represents the mean gain in quality-adjusted life years per participant in the base case analysis.

As reported in Bennett et al, mean difference in weight change of the intervention and UC arms with regard to baseline approached statistical significance at 6 months (−1.1 kg [95% CI −2.3 to 0.04]), and was statistically significant at the 12-month (−1.4 kg [−2.8 to −0.1]) and 18-month (−1.7 kg [−3.3 to −0.2]) assessments [18]. The difference in weight change across arms was transformed to QoL change scores for Shape participants and UC participants at 6 months (+0.009 and +0.006, respectively), 12 months (+0.009 and +0.005, respectively), and 18 months (+0.009 and +0.004, respectively) from baseline. Figure 1 presents the graph of QoL change against time from baseline.

#### Base-Case Incremental Cost-Effectiveness Ratio

In the base case of a 1-year intervention followed by 5 years of linear decay of post-trial weight gain prevention benefits, we estimated an ICER of US $55,264 per QALY gained.

#### Sensitivity Analyses

**Figure 2:**
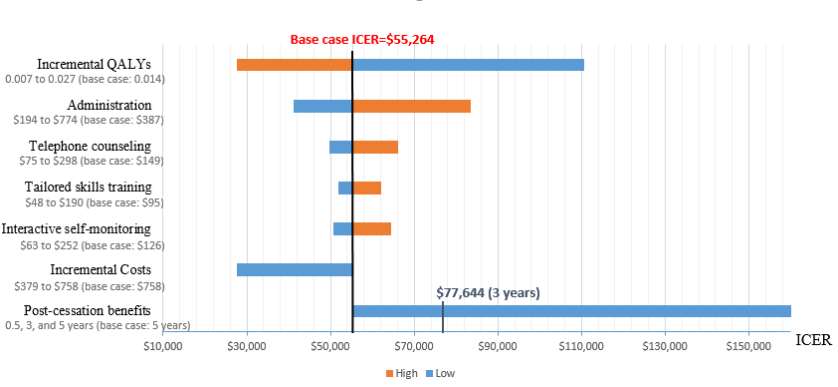
Results of one-way sensitivity analyses varying key parameters (incremental QALYs, incremental and category-specific costs, and duration of post-cessation benefits). ICER: incremental cost-effectiveness ratio; QALYs: quality-adjusted life years.

One-way sensitivity analyses showed that halving incremental QALYs of the intervention arm with regard to UC raised the ICER to US $110,529 per QALY, whereas doubling incremental QALYs or halving incremental costs decreased the ICER to US $27,632 per QALY. When each Shape cost category was doubled or halved separately, the ICER ranged from US $41,130 to US $83,447 (administration costs); US $49,792 to US $66,122 (telephone counseling costs); US $51,766 to US $62,176 (tailored skills training costs); and US $50,637 to US $64,434 (interactive self-monitoring costs). With QoL benefits modeled to decay to zero within 3 years of cessation of the intervention, the ICER was US $77,644; with benefits ceasing 6 months after the intervention concluded, the ICER rose to US $165,730. Figure 2 presents a tornado diagram showing the results of the one-way sensitivity analyses.

#### Probabilistic Sensitivity Analyses

**Figure 3:**
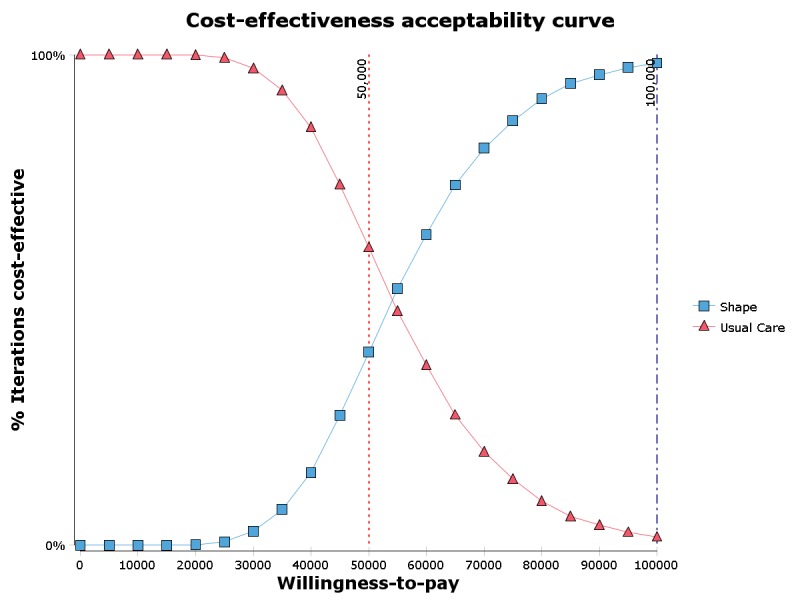
Cost-effectiveness acceptability curve compared against 2 potential cost-effectiveness thresholds.

Figure 3 displays the cost-effectiveness acceptability curve. The figure reveals that 39.3% of simulations suggest that the incremental cost per QALY of Shape relative to UC is less than US $50,000, an oft-cited threshold for cost-effectiveness [28,29]. At a willingness to pay of US $100,000 per QALY (another commonly cited threshold), 98.3% of simulations suggested that Shape is cost-effective [30,31].

## Discussion

### Principal Findings

This study presents the first evidence that a digital weight management program can be a cost-effective solution for preventing weight gain. In our base case, we estimated the incremental cost-effectiveness of Shape per QALY gained to be US $55,264, slightly higher than the often-quoted threshold of US $50,000 per QALY. The reality is that there is no established threshold for cost-effectiveness of health care interventions in the United States; indeed, the Patient Protection and Affordable Care Act specifically bars the use of a cost-effectiveness measure as a threshold [32]. Despite this, a threshold of US $50,000 per QALY has been widely used in the United States since 1992 [29]. In probabilistic sensitivity analyses, 39% of our simulations suggested that Shape has an ICER below this threshold. Some researchers even suggest a more appropriate threshold would be US $100,000 per QALY [30,31]. Compared against this threshold, 98% of our simulations suggest that Shape is cost-effective. Although there are no other weight gain prevention programs to compare against, Shape’s ICER also compares favorably to the majority of the lifestyle and pharmacological interventions targeting weight loss [33]. Moreover, although this study did not quantify cost offsets from slower weight progression, Cawley et al suggest that annual savings from even moderate weight loss (or less weight gain relative to control) far exceed the US $758 annual cost of the program [34]. Moreover, there are several reasons to believe Shape’s estimated cost of US $758 is likely to be an upper bound. Costs in years 3 to 5, when the program was recruiting at a much higher rate, were an average of 35% lower than that of the first 2 years (US $11,380 in years 3 to 5 compared with $17,401 in years 1 to 2; Table 1). If one considers only the per participant variable costs of the pedometer, scale, skills training kit bag, and coaching time, with the remaining costs averaged over a very large number of participants, per capita costs could be as low as US $243 (see Multimedia Appendix 1). This suggests that at full scale, Shape may be highly cost-effective. However, it may be that costs would need to be further reduced with no loss in outcomes for Shape to be highly scalable. For example, Weight Watchers OnlinePlus costs only US $160 per year [35]. Shape may need to demonstrate an average cost per participant in this range or better to increase the potential for scalability. This could be accomplished through better use of Shape’s data to customize the intervention at the individual level and to intervene early for those most at risk of dropping out. This should be an area of future research.

### Limitations

Although this study has many strengths, we identified 5 key limitations in this study. First, there is a lack of evidence on the persistence of weight gain prevention effects post intervention. The existing literature strongly suggests significant weight loss maintenance, and presumably QoL gains, up to 5 years after successful weight loss programs [36,37]. However, there are no corresponding data for weight gain prevention. We made the assumption that quality-of-life benefits would last 5 years beyond the intervention period and tested the sensitivity of the ICER to this assumption. Sensitivity analyses suggested that the ICER was highly sensitive to the duration over which benefits persist. Studies with longer-term follow-ups of both weight loss and QoL are required to validate our assumption in the context of weight gain prevention programs. Second, owing to data limitations, we were not able to directly assess QoL in participants in this study. Instead, we used an approach similar to that used in other cost-effectiveness studies [26,27] and assessed the sensitivity of our estimates to uncertainty in the QoL estimates using one-way and probabilistic sensitivity analyses. Analyses suggest that the ICER is moderately sensitive to the relationship between QoL and weight change. Third, estimates of QoL changes are sensitive to the method of elicitation. This is true whether one uses direct elicitation methods, such as standard gamble or time-tradeoff methods, or using patient-reported outcomes measures, such as the data from the SF-12 version 2 instrument used in this study [38]. This fact, combined with our imputation strategy which imputes QoL changes solely from weight change and age, ignoring other potentially important confounders, suggests that there is likely a high degree of error in our QoL estimates, as would be the case in most cost-effectiveness studies. We address this via sensitivity analyses using a wide range of QoL values that we believe capture reasonable lower and upper bounds for these estimates. Fourth, the trial did not measure potential cost offsets from reduced health care utilization that may result from improved participant health outcomes. As a result, cost-effectiveness results presented here may be conservative. Finally, this program was delivered to black women in a low-income rural community health center setting. Although the intervention could be fielded in any setting and to diverse populations, future studies would be needed to see if the results are generalizable. However, long-term studies that follow a cohort of participants over an extended period of time and link their weight loss to changes in health care utilization would be needed to truly confirm the long-term cost-effectiveness of the Shape intervention.

### Conclusions

Although long-term studies are needed to confirm this result, this study suggests that Shape is likely to be a cost-effective intervention to prevent weight gain and reduce risks for chronic disease among high-risk black women in low-income rural communities, where obesity rates are also highest. It thus provides an additional strategy that these communities can rely on to effectively and efficiently respond to the nation’s growing obesity epidemic.

## Appendix

Multimedia Appendix 1 Costs of Shape Program by Program Activity (all costs in 2018 US $).

## Abbreviations

BMI: body mass index
ICER: incremental cost-effectiveness ratio
QALY: quality-adjusted life year
QoL: quality of life
UC: usual care
