# Supplementary material for: A Digital Behavioral Weight Gain Prevention Intervention in Primary Care Practice: Cost and Cost-Effectiveness Analysis
Source: J Med Internet Res. 2019 May 17;21(5):e12201. doi: 10.2196/12201 (PMC6543798; doi:10.2196/12201)
Supplement: Multimedia Appendix 1 [file jmir_v21i5e12201_app1.pdf]

Appendix:

Costs of Shape Program by Program Activity (all costs in 2018 USD)

	Y1	Y2	Y3	Y4	Y5
**Self Monitoring**					
IVR System	$540	$523	$505	$489	$480
Pedometers	$228	$0	$0	$0	$0
Scales	$5,724	$0	$0	$0	$0
Domain Name	$13	$18	$18	$17	$17
Joyent server	$619	$599	$579	$561	$550
**Skills Training**					
Print Materials (curriculum)	$296	$4,585	$2,161	$132	$0
Lunch bags	$343	$0	$0	$0	$0
Water bottle	$229	$0	$0	$0	$0
Gym bag	$916	$0	$0	$0	$0
Video Production	$0	$0	$0	$0	$0
**Telephone Counseling**					
Health Educator/Dietitian (1 FTE)	$0	$1,954	$1,889	$1,830	$1,794
Coach Training	$220	$0	$0	$0	$0
Motivational Interviewing Videos	$336	$0	$0	$0	$0
Coach Binders	$377	$0	$0	$0	$0
Coach Cell Phone Plan	$0	$1,462	$1,413	$1,369	$0
Coach Netbook	$944	$0	$0	$0	$0
Technology Build	$0	$0	$0	$0	$0
**Administration**					

Personnel					
Public Program Manager (0.25 FTE)	$6,816	$6,601	$6,378	$6,180	$6,060
Training and Equipment					
Support Staff Training	$220	$0	$0	$0	$0
Overheads (Space, Lights, Computer, Internet Access)	$629	$609	$589	$570	$559
